# Seasonal and intracanopy shifts in the fates of absorbed photons in central Amazonian forests: implications for leaf fluorescence and photosynthesis

**DOI:** 10.1111/nph.70183

**Published:** 2025-05-29

**Authors:** Leonardo G. Ziccardi, David Kramer, Nathan Gonçalves, Bruce W. Nelson, Tyeen Taylor, Loren P. Albert, Kleber S. Campos, Neill Prohaska, Natalia Restrepo‐Coupe, Scott R. Saleska, Scott C. Stark

**Affiliations:** ^1^ Department of Forestry Michigan State University East Lansing MI 48824 USA; ^2^ Program in Ecology, Evolution, and Behavior Michigan State University East Lansing MI 48824 USA; ^3^ Department of Energy‐Plant Research Laboratory Michigan State University East Lansing MI 48824 USA; ^4^ Department of Biochemistry and Molecular Biology Michigan State University East Lansing MI 48824 USA; ^5^ Jan IngenHousz Institute Wageningen 6708 PE the Netherlands; ^6^ Brazil's National Institute for Amazon Research (INPA) Manaus AM 69067‐375 Brazil; ^7^ The Operator's Manual for the Planet (The OMP, LLC) Gustavus 99826 UK; ^8^ Department of Forest Ecosystems and Society Oregon State University Corvallis OR 97330 USA; ^9^ Department of Environmental Physics Federal University of Western Pará (UFOPA) Santarém PA 68040‐255 Brazil; ^10^ Department of Ecology and Evolutionary Biology University of Arizona Tucson AZ 85721 USA

**Keywords:** Amazonia, Brazil, canopy microenvironments, solar‐induced fluorescence (SIF), tropical forest

## Abstract

- Recent studies have shown a linear relationship between solar‐induced Chl fluorescence (SIF) and gross primary productivity (GPP) at large scales. However, this relationship diverges at finer leaf scales, particularly in tropical forests with complex canopy structures.
- To address this issue, we assessed seasonal and intracanopy variations in leaf energy partitioning in central Amazonian forests with extensive in‐canopy sampling and pulse‐amplitude‐modulated Chl fluorescence measurements. We explored the pathways of photon utilization for photochemistry (ΦPSII), heat dissipation (ΦNPQ), and nonregulated quenching (ΦNO) of fluorescence.
- We found consistent increases in ΦNPQ and decreases in ΦPSII and ΦNO with increasing canopy height, primarily driven by changes in photosynthetically active radiation. During the dry season, a triphasic relationship between ΦNO and ΦPSII was detected, alternating between positive and negative relationships across leaf irradiance levels, highlighting stress‐induced physiological responses. Interspecific variation and vapor pressure deficit also played significant roles in modulating ΦNO, emphasizing the complex interaction between environmental factors, species composition, and energy dissipation across canopy strata.
- These insights into leaf‐level fluorescence and energy dynamics show the complex mediation of ΦNPQ‐ΦNO‐ΦPSII relationships, offering implications for enhancing SIF‐GPP relationships and understanding tropical forest responses to climate change.

## Abstract 2

See also the Commentary on this article by Sun *et al.* **248**: 5–7.

## Resumo

- Estudos recentes demonstram uma relação linear entre a fluorescência da clorofila induzida por luz solar (SIF) e a produtividade primária bruta (GPP) em largas escalas. No entanto, essa relação diverge a nível de folha em escalas mais finas, particularmente em florestas tropicais com dosseis estruturalmente complexos.
- Para abordar essa questão, avaliamos variações sazonais e verticais na partição de energia nas folhas em florestas da Amazônia Central, utilizando uma amostragem extensiva de folhas ao longo do perfil do dossel e medições de fluorescência da clorofila com modulação de amplitude de pulso (PAM). Exploramos as diferentes rotas de utilização dos fótons para a fotossíntese (ΦPSII), dissipação térmica (ΦNPQ) e quenching não regulado (ΦNO) da fluorescência.
- Observamos aumentos consistentes em ΦNPQ e reduções em ΦPSII e ΦNO com o aumento da altura no dossel, impulsionados principalmente por variações em radiação fotossinteticamente ativa (PAR). Durante a estação seca, foi detectada uma relação trifásica entre ΦNO e ΦPSII, alternando entre correlações positivas e negativas ao longo dos níveis de irradiância foliar, destacando respostas fisiológicas induzidas por estresse. A variação interespecífica e o déficit de pressão de vapor (VPD) também desempenharam papéis significativos na modulação de ΦNO, enfatizando a complexa interação entre fatores ambientais, composição de espécies e dissipação de energia ao longo dos estratos do dossel.
- Esses insights sobre fluorescência foliar e a dinâmica energética evidenciam uma complexa mediação das relações ΦNPQ–ΦNO–ΦPSII, oferecendo implicações para o aprimoramento das relações SIF‐GPP e para a compreensão das respostas das florestas tropicais às mudanças climáticas.

## Introduction

Plant photosynthesis is the primary driver of terrestrial carbon uptake that determines ecosystem gross primary productivity (GPP). Tropical rainforests, which store nearly half of global forest carbon and play a vital role in regulating global carbon and water cycles, are increasingly threatened by extreme droughts and heatwaves and may potentially turn into carbon sources due to increased tree mortality (Bonan, 2008; Phillips *et al*., 2009; Pan *et al*., 2011; Brienen *et al*., 2015; Aleixo *et al*., 2019; Chen *et al*., 2024). However, uncertainty in models of carbon uptake remains significant in the tropics, disrupting accurate predictions of ecosystem responses to environmental change (Huntzinger *et al*., 2017; L. Liu *et al*., 2024; Melnikova *et al*., 2024). Highly accurate remote observation of photosynthetic activity that can be linked to physiological and ecological timescales over which production varies in tropical forests has been a major goal of predictive Earth Systems Science.

The discovery that GPP could be remotely estimated by satellite measurements of solar‐induced Chl fluorescence (SIF) – a signal spanning the red and far‐red spectral region (*c*. 650–800 nm) emitted by leaves as a by‐product of the light reactions of photosynthesis – led to a flurry of studies capitalizing on this promising proxy for observing ecosystem function (Mohammed *et al*., 2019). This advancement, achieved by decades of research (Krause & Weis, 1991; Zarco‐Tejada *et al*., 2000; Moya & Cerovic, 2004; Plascyk & Gabriel, 2007; Rossini *et al*., 2010; Frankenberg *et al*., 2011; Joiner *et al*., 2011), has revolutionized our ability to track the terrestrial carbon cycle from space. The basic theory underlying SIF is that the light‐harvesting complexes of photosystems absorb photons and then channel this energy either into the light reactions of photosynthesis, into a heat dissipation pathway, or into re‐emitted fluorescence. The latter process generates the signal detected as SIF. Unlike reflectance‐based metrics (vegetation indices), which infer canopy photosynthetic capacity rather than rate, SIF offers a more direct measure of canopy productivity by capturing a signal (fluorescence) linked to actual photosynthetic activity (Doughty *et al*., 2019). However, for SIF to fulfill its potential as a metric for GPP, a consistent relationship must be established at the leaf level between the yields of absorbed photons used for photosynthesis and those re‐emitted as fluorescence. This relationship becomes nonlinear when environmental stressors such as high temperatures, low soil water availability, and strong light saturation come into play (Porcar‐Castell *et al*., 2014; Van der Tol *et al*., 2014; Martini *et al*., 2022).

The temporal and spatial scale dependencies of the SIF and GPP relationship remain a subject of debate (Bandopadhyay *et al*., 2020; Zhang *et al*., 2023; Qiu *et al*., 2024). Despite rapid advancements in SIF measurement technology, our ability to interpret the acquired data at the tropics remains limited (e.g. Porcar‐Castell *et al*., 2021). While satellite‐observed SIF has been shown to correlate with GPP estimates at many sites (Guanter *et al*., 2014; Sun *et al*., 2017; Li *et al*., 2018b; Magney *et al*., 2019), the relationship between SIF and photosynthesis can vary by *c*. 60% across different biomes and environmental conditions (Li *et al*., 2018b). While the majority of studies have focused on temperate forests, there is limited direct evidence for accurate SIF‐GPP relationships in tropical forests (Zhang *et al*., 2023). Understanding key factors that can be used to improve this relationship in dense and structurally complex canopies is essential for evaluating vegetation responses to climate change at large scales.

Inferences of GPP from SIF can be improved somewhat by empirical corrections based on plant physiological responses to environmental variation (Verma *et al*., 2017; Li *et al*., 2018a; Yu *et al*., 2019). Thus, a deeper understanding of the physiological and environmental drivers of this relationship would produce more robust insights from SIF (Magney *et al*., 2020), especially in remote regions such as tropical forests where eddy flux towers for GPP validation are scarce. Mechanisms of fluorescence relationships to photosynthesis can be studied with pulse‐amplitude‐modulated (PAM) fluorometry, a technique used to quantify the pathways of energy absorption by photosystems, including fluorescence, photosynthesis, and nonphotochemical quenching (NPQ), in which energy is dissipated as heat (Hendrickson *et al*., 2004; Kramer *et al*., 2004). The passive detection of fluorescence, such as SIF measured from above‐canopy towers or satellites, does not provide direct estimates of these quantum yields (Porcar‐Castell *et al*., 2014; Van der Tol *et al*., 2014; Magney *et al*., 2019; Maguire *et al*., 2020).

As an active sensor leaf‐level system, PAM fluorometry has been extensively employed to measure Chl *a* fluorescence (ChlF, measured in units of μmol m^−2^ s^−1^) and quantify the fluxes of photons through photochemical and nonphotochemical pathways after absorption by photosystem II (PSII). These include the yield of photochemistry (ΦPSII), the yield for dissipation through downregulation (ΦNPQ), and the yield of other nonphotochemical losses (ΦNO) (Hendrickson *et al*., 2004; Kramer *et al*., 2004). ΦNO is the fluorescence yield (ΦF) plus the basal heat dissipation (ΦD). Thus, ΦNO reflects nonlight‐induced (‘basal’ or ‘dark’) quenching processes and represents the fluorescence yield (ΦF) under steady‐state conditions approached during measurement (Hendrickson *et al*., 2004; Kramer *et al*., 2004; Klughammer & Schreiber, 2008). As ΦNO is not arbitrarily scaled by Chl content and leaf optical properties (Mattila & Tyystjärvi, 2023; Urban *et al*., 2023), it is a robust parameter for comparing quantum yield dynamics of distinct species and facilitating cross‐instrument inferences (Helm *et al*., 2020).

Previous studies have demonstrated a strong nonlinearity in the relationship between ΦF and ΦPSII, in which the regression oscillates between positive and negative across leaf irradiance levels (Van der Tol *et al*., 2014; Maguire *et al*., 2020). This nonlinearity, influenced by heat dissipation through ΦNPQ (Porcar‐Castell *et al*., 2014; Martini *et al*., 2022), indicates that SIF yield alone, or its close approximation at the leaf level via ΦNO (Helm *et al*., 2020), is an insufficient proxy for assessing photosynthetic status. It is important to note that, at broader scales, SIF is predominantly influenced by the absorbed photosynthetically active radiation (APAR). Increased Chl content typically correlates with higher APAR, and consequently, greater SIF is associated with dense forest canopies in tropical regions (Doughty *et al*., 2021a). However, in tropical forests, APAR is not only linked to leaf physiological traits that determine leaf‐level light absorption but also to seasonal changes in canopy structure. These changes arise from variations in leaf area, leaf angle, and Chl concentration from phenological processes, affecting the net SIF emitted from leaves at different heights (Vilfan *et al*., 2016; Doughty *et al*., 2019; Castro *et al*., 2020). Therefore, while the leaf‐level understanding of energy partitioning pathways is essential to enhance local and regional inferences, they do not solely define SIF‐GPP relationships at larger scales. Seasonal variations in light and soil water availability may also affect the ΦPSII‐ΦF relationship at the leaf, likely due to sustained NPQ or the photoinhibition of reaction centers under conditions of environmental stress (Porcar‐Castell *et al*., 2014). For example, a previous study found that ΦF can increase even when the photochemical yield fraction drops below *c*. 0.3 during periods of high‐light stress and elevated NPQ (Van der Tol *et al*., 2014); this could invert the relationship between true and expected variation in GPP when interpreted by SIF.

In tropical ecosystems, canopy structure plays a critical role in shaping microenvironmental variations that affect the relationship between ChlF and photosynthesis (Köhler *et al*., 2018b; Pinagé *et al*., 2022). The complexity of dense tropical canopies creates conditions for the occurrence of subcanopy fractions of sunlit and shaded foliage simultaneously on days of high top‐of‐canopy irradiation (Li *et al*., 2023). In this context, leaf‐level understanding of asymmetric responses of photosynthesis and fluorescence to irradiance levels along the canopy profile can be used to generate empirical correction for full‐sun and overcast satellite SIF observations, improving GPP prediction in future models (Regaieg *et al*., 2021).

Studies in crop fields, glasshouses, and temperate forests have explored the connection between ChlF, photosynthetic function, and environmental variability, providing insights into how these relationships can be interpreted in tower and satellite SIF observations (Van der Tol *et al*., 2014; He *et al*., 2020; Maguire *et al*., 2020; Marrs *et al*., 2020; Chang *et al*., 2021). However, while many investigations focused on understanding the regulation of photosynthesis and photoprotection in response to environmental variation (Demmig‐Adams *et al*., 1996; Hendrickson *et al*., 2004; Kramer *et al*., 2004; Klughammer & Schreiber, 2008; Schreiber & Klughammer, 2008; Santos *et al*., 2019), the influence of canopy structure on seasonal variations of leaf energy partitioning in tropical forests is frequently ignored. Understanding how microenvironments associated with canopy structure mediates these processes is essential for improving our interpretation of SIF as a proxy for GPP across scales (Gu *et al*., 2019).

This study aimed to fill a critical knowledge gap to understand how photons absorbed by leaves are partitioned over environmental gradients of complex tropical forest canopies, motivated by the dual needs to advance understanding of the response of photosynthesis to climate variation and accurate inferences on forest productivity from SIF observations. By conducting *in vivo* measurements using PAM ChlF across vertical forest profiles, we first examine how canopy environments affect the fluxes of absorbed photons through photochemical (ΦPSII) and nonphotochemical (ΦNO and ΦNPQ) pathways. Given that changes in canopy structure, air temperature, and atmospheric water deficit also affect seasonal changes in the light responses of SIF and GPP (Raczka *et al*., 2019; Green *et al*., 2020; Pierrat *et al*., 2022), we then assess the effects of instantaneous PAR, vapor pressure deficit (VPD), and height on these quantum yields across seasons. By linking leaf energy partitioning to height, our findings can be integrated with leaf area density profiles (e.g. Stark *et al*., 2012; Smith *et al*., 2019) to improve SIF simulations based on forest structure in radiative transfer models (i.e. Malenovský *et al*., 2021; Gao *et al*., 2022; Jin *et al*., 2024). Finally, based on previous studies conducted in boreal and temperate regions (e.g. Porcar‐Castell *et al*., 2014; Maguire *et al*., 2020; Pierrat *et al*., 2024), we identify the breakpoints of the nonlinear relationship between ΦNO and ΦPSII driven by irradiance at the forest level. Our findings highlight a triphasic relationship emerging during the dry season, in which photosynthesis saturates at high light and steady‐state fluorescence increases in response to stress. By offering novel insights into seasonal and intracanopy leaf energy dynamics and their implications, this study contributes to enhancing SIF‐based ecosystem GPP predictions in tropical forests.

## Materials and Methods

### Study site

The research was conducted at the Tapajós National Forest (TNF) site, located near 67 km (K67, 02°51′S, 54°58′W) of the Santarém–Cuiabá highway, south of the city of Santarém (Pará, Brazil). It is an evergreen tropical forest on a well‐drained infertile oxisol plateau, with a mean canopy height of *c*. 40–45 m and a subdominant layer ranging from 15 to 30 m (with a mean of 28 m) (Hutyra *et al*., 2007; Hunter *et al*., 2015). This region shows the longest dry season for dense closed‐canopy forests in Amazonia (5 months with < 100 mm rainfall) (Espinoza Villar *et al*., 2009).

### Seasonal sampling with PAM measurements

In order to cover seasonal variation of leaf microenvironments and energy partitioning, we compared measurements between dry (July–December) and wet (January–March) seasons in the TNF K67 site. Dry season field campaigns were conducted in 2019, 2021, and 2022, and a wet season campaign was conducted in 2023. Pulse‐amplitude‐modulated ChlF measurements and sampling of microenvironmental variation at the leaf level were conducted with the MultispeQ (v.2.0), a portable instrument equipped with two photodiode detectors covering the visible spectral range (400–700 nm) and the near infrared (700–1150 nm), capable of measuring environmental, fluorescence‐based, and absorbance‐based parameters (see Kuhlgert *et al*., 2016, for measurement theory and detailed instrument description). The MultispeQ replicates ambient photosynthetically active radiation (PAR) inside a leaf chamber, enabling quick measurements (*c*. 30 s) of leaf‐level PAR (in μmol m^−2^ s^−1^), air temperature (°C), relative humidity (%), ΦNO, ΦPSII, and ΦNPQ while photosynthesis remains active. The parameter ΦNO, which combines nonregulated quantum yields including ΦF, correlates more closely with leaf‐level SIF yield measured by passive instruments than steady‐state fluorescence alone, as observed by Helm *et al*. (2020). Leaf‐level VPD was derived from relative humidity and air temperature values recorded at the moment of each physiological measurement. The MultispeQ thus enables the capture of ChlF and environmental parameters that represent near‐instantaneous, *in situ* values. From Tietz *et al*. (2017), ΦNO was calculated as follows:

(Eqn 1) $$\mathrm{\Phi NO}=\frac{1}{1+\mathrm{qL}\times 4.88+\text{NPQt}}$$

where qL is an estimate of the fraction of PSII centers in open state based on a ‘lake’ model for the photosynthetic unit (Kramer *et al*., 2004). NPQt is a parameter used for rapid estimation of NPQ without requiring the relative fluorescence yield value (*F*_m_) obtained during a saturation pulse in dark‐adapted leaves, where NPQ is assumed to be zero. This makes NPQt well‐suited for measurements under steady‐state illumination in field conditions (Tietz *et al*., 2017):

(Eqn 2) $$\text{NPQt}=\frac{4.88}{\left(F_{m}^{\prime }/F_{o}^{\prime }\right)-1}-1$$

where $F_{m}^{\prime }$ is the maximum fluorescence under steady‐state illumination, and $F_{o}^{\prime }$ is the relative fluorescence yield under steady‐state illumination after full oxidation of *Q*_A_ by far‐red light (Kuhlgert *et al*., 2016; Tietz *et al*., 2017). Then, ΦPSII is calculated based on the lake model as follows:

(Eqn 3) $$\text{ΦPSII}=\frac{F_{m}^{\prime }-F_{s}}{F_{m}^{\prime }}$$

where *F*_s_ is the relative steady‐state fluorescence yield measured by a modulated fluorometer. Considering that the sum of photochemical and nonphotochemical yields must be unity, ΦNPQ can be calculated as follows:

(Eqn 4) ΦNPQ=1−ΦPSII−ΦNO

Previous studies have demonstrated the importance of accurately accounting for qL in the mechanistic models of GPP derived from remote sensing SIF observations (Gu *et al*., 2019; Z. Liu *et al*., 2024). Open (or reduced) PSII reaction centers transition into a close (or oxidized) state as electrons are transferred downstream to the plastoquinone pool. While PAR is well‐established as a key driver of qL variations (Kramer *et al*., 2004; Baker, 2008; Gu *et al*., 2019), predictions of qL across leaf and canopy scales remain in their early stages due to a limited comprehensive understanding of qL and NPQ responses to additional environmental factors (Z. Liu *et al*., 2024).

**Fig. 1:**
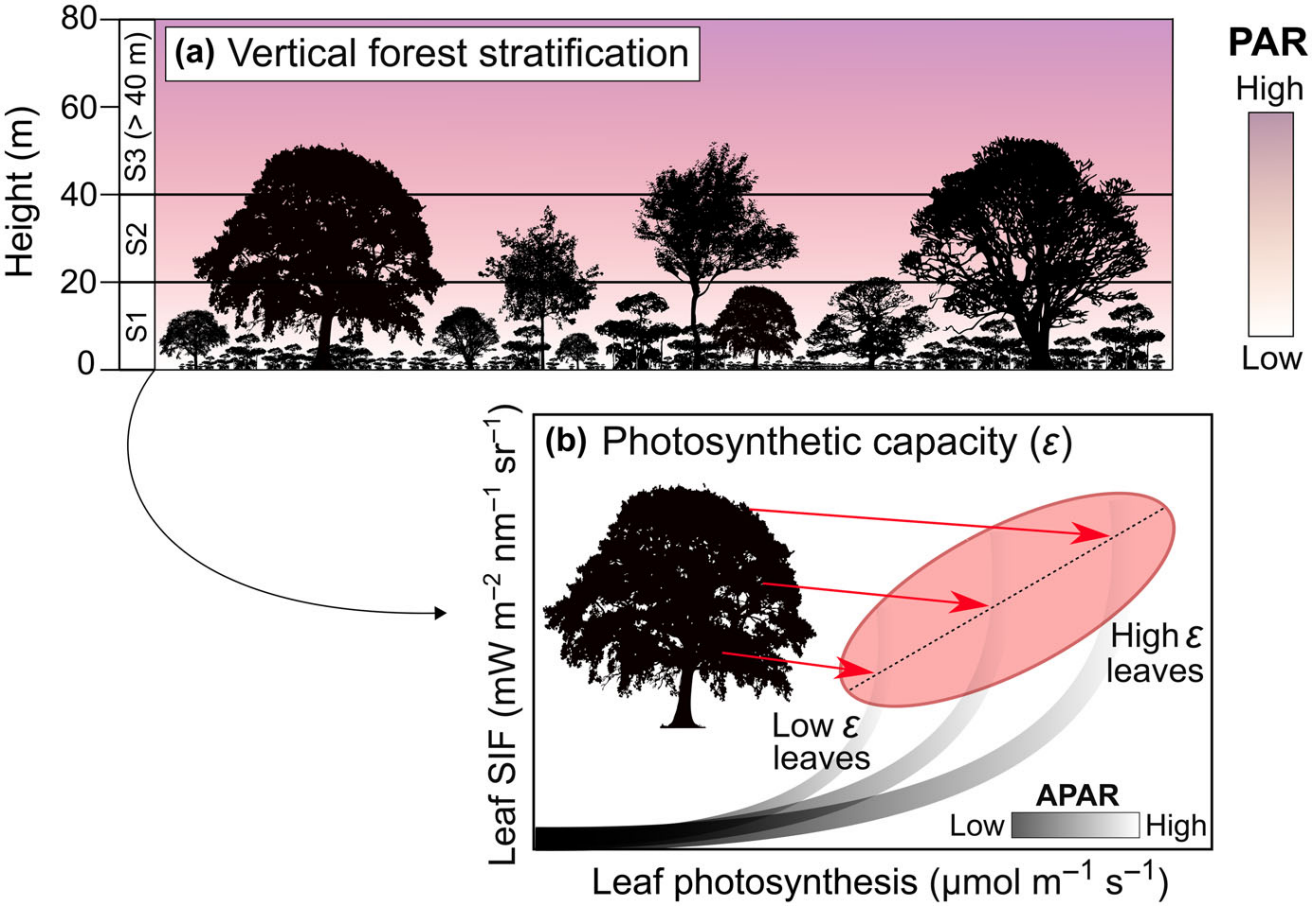
(a) Forests vertical stratification illustrating variation in photosynthetically active radiation (PAR; in μmol m^−2^ s^−1^). (b) Individual‐level sampling approach using advanced tree climbing techniques to access different canopy levels and capture variations in absorbed PAR (APAR) by measuring *in situ* leaf chlorophyll fluorescence at three different crown positions with the MultispeQ (adapted from Magney *et al*., 2020). SIF, solar‐induced Chl fluorescence.

Canopy environments were sampled vertically according to a simple scheme that defined three strata based on height from the ground. We utilized technical climbing to sample leaves *in situ* from locally dominant tree species in each canopy stratum in terms of aboveground biomass, following ongoing forest inventories conducted at the K67 site since 1999 (Pyle *et al*., 2008). Leaves fully expanded, mature, free of epiphylls, and lacking necrotic and chlorotic patches were measured from the lower, middle, and upper regions of the crown to provide a representative sample. Three vertical forest layers were defined as the lower canopy stratum (S1: 0–20 m); the mid‐canopy stratum (S2: 20–40 m); and the upper canopy stratum (S3: > 40 m). The first threshold at 20 m was based on a previous ground‐based light detection and ranging (LiDAR) study at the TNF, which identified this height as the cutoff in which the lower and upper canopy levels exhibited distinct seasonal leaf area responses (Smith *et al*., 2019; Figs 1, 3). The 40‐m threshold was informed by Hutyra *et al*. (2007), who found it to be approximately the average tall upper canopy height at our TNF site. Additionally, our observations suggest that heights above 40 m are largely restricted to emergent trees. This division allowed us to examine the unique dynamics of functional niches represented by each canopy stratum (Fig. 1a). To provide a representative sample of leaves exposed to both low and high irradiance regimes, we sampled three branches per tree at different crown positions: one from the bottom, one from the middle, and one from the top regions (Fig. 1b), with at least three leaves measured on each branch.

To ensure data quality, we excluded observations with noisy raw measurements, which may have resulted from involuntary movement during measurements or insufficient foliage in the instrument viewing chamber. This filtering was based on the criterion that the combined values of ΦPSII, ΦNO, and ΦNPQ should sum to 1, with each individual value being greater than zero. After this filtering, the final dataset consisted of 3121 observations, with 1237 observations from 14 locally dominant species in the wet season and 1884 observations from 18 locally dominant species in the dry season (Supporting Information Table S1). One unidentified tree species was sampled during the dry season in S2 and S3. We used a measuring tape extending to the ground to determine the height of each sampled leaf.

### Data analysis

To examine variations of ΦNO, ΦPSII, and ΦNPQ across canopy strata, we compared the vertical canopy profiles generated by leaf‐level measurements with the MultispeQ across seasons. We calculated the average relative proportions of quantum yields in each canopy stratum and conducted tests to determine significant differences among strata. Differences among canopy strata were assessed through the nonparametric Kruskal–Wallis test, followed by the Mann–Whitney *U* test (Mann & Whitney, 1947). To observe intracanopy variations in PAR, VPD, and the three quantum yields across the vertical forest profile, mean values and associated confidence intervals were calculated for each variable, stratified by 1‐m height increments. Furthermore, we utilized the mgcv package in R (Wood, 2001) to fit generalized additive models (GAMs) and examine the relationship between each quantum yield and PAR across the vertical canopy profile in each season.

To investigate the effects of instantaneous PAR, VPD, and height on the yields for energy dissipative processes, we employed generalized mixed multiple regression models (GMMs). We fitted separate models for each quantum yield (ΦNO, ΦPSII, and ΦNPQ), season (dry and wet), and canopy stratum (S1, S2, and S3), including species as a random effect. The microenvironmental predictors used in the models were PAR, VPD, and height. This approach allowed us to model the influence of microenvironmental variables while accounting for interspecific autocorrelation. We calculated standardized confidence intervals for each fixed effect and considered a predictor to have a significant effect in the model when these intervals did not overlap zero.

(Eqn 5) $$Y_{0}=\beta 0+\beta 1\left(\mathrm{PAR}\right)+\beta 2\left(\mathrm{VPD}\right)+\beta 3\left(H\right)+f\left(\alpha \times \mathrm{sp}\right)+\varepsilon$$

where $Y_{0}$, quantum yields (ΦPSII; ΦNO; ΦNPQ); *β*0, intercept; *β*1, *β*2, and *β*3, coefficients for the corresponding predictor variables; PAR, photosynthetically active radiation (in μmol m^−2^ s^−1^); VPD, vapor pressure deficit (in kPa); *H*, height (in m); *α*, the effect of the random factor for species (sp); *ε*, the error term capturing unexplained variance in the model.

We tested for multicollinearity of the predictors in each model through the variance inflation factor (VIF), using the *vif* function from the car package in R (Fox *et al*., 2012). We observed VIF values below 2 for all predictors in all models (Table S2), indicating low multicollinearity and reliable assessment of relative effects. To determine the explanatory value of these models, we used the R package piecewisesem to calculate marginal and conditional adjusted *R* ^2^ values (Lefcheck , 2015). Marginal *R* ^2^ values were used to assess the extent to which environmental variables (fixed effect) explained the variability in photochemical and nonphotochemical yields (Nakagawa & Schielzeth, 2013). The influence of species (random effect) was evaluated by the difference between conditional and marginal *R* ^2^ values. While this approach enabled us to assess the effects of environmental drivers and interspecific variation on ΦPSII, ΦNO, and ΦNPQ within each canopy stratum, we further employed generalized additive mixed effect models (GAMMs) to explore how PAR and VPD influence these quantum yields across seasons at the forest level. GAMMs were fitted using a beta distribution with a maximum likelihood estimation of parameters, also considering each yield independently. These models included soothed terms modeled by season for PAR, VPD, and height, while also considering season as a categorical predictor and species as a random effect:

(Eqn 6) $$Y_{0}=f\left(\mathrm{PAR},\mathrm{by}=S\right),f\left(\mathrm{VPD},\mathrm{by}=S\right),f\left(H,\mathrm{by}=S\right),S+f\left(\alpha \times \mathrm{sp}\right)+\varepsilon$$

where *Y*_0_, quantum yields (ΦPSII; ΦNO; ΦNPQ); *f*, a smooth function modeled using regression splines for predictor variables, with *k* = 5 basis functions controlling smoothness; PAR, photosynthetically active radiation (in μmol m^−2^ s^−1^); VPD, vapor pressure deficit (in kPa); *H*, height (in m); *S*, seasons, with two levels (wet and dry); *α*, the effect of the random factor for species (sp); *ε*, the error term capturing unexplained variance in the model.

Seasonal differences in smooth effects were observed using the *plotDiff* function from the mgcviz package in R (Fasiolo *et al*., 2020). We used the R package gam.hp to assess the hierarchical partitioning of each predictor to the explained deviance in our GAMMs (Lai *et al*., 2024). To assess nonlinearities in the relationship between ΦNO and ΦPSII, we fitted separate GAMs for these variables for the wet and dry seasons. Breakpoints in the relationship between ΦNO and ΦPSII (the points at which there is a change in this relationship) were identified by calculating the derivatives of the GAMs and determining where the slope switched from positive to negative, following Maguire *et al*. (2020). We then compared the ΦPSII breakpoints and the PAR values associated with each breakpoint between the dry and wet seasons. To assess the performance of different GAMs, we used the Akaike information criterion (AIC), which measures the trade‐off between model fit and complexity. By comparing the AIC, we evaluated whether GAMs with restricted knots or unrestricted knots better capture the variations in the relationship between ΦNO and ΦPSII. Based on this evaluative criterion (e.g. Burnham & Anderson, 2004), we selected models without any restrictions on the number of knots for both the dry and wet seasons. We also evaluated the effective degrees of freedom (edf) of each model. The edf reflects the degree of nonlinearity of a curve, in which values around one indicate a close‐to‐linear relationship (Wood, 2006). Finally, to expand the applicability of the mechanistic light reaction model proposed by Gu *et al*. (2019) to tropical forests, we examined intracanopy variations in qL and NPQt, along with their seasonal relationships with PAR and VPD (Methods S1). We conducted all analyses in the R software (R Core Team, 2023).

## Results

**Fig. 2:**
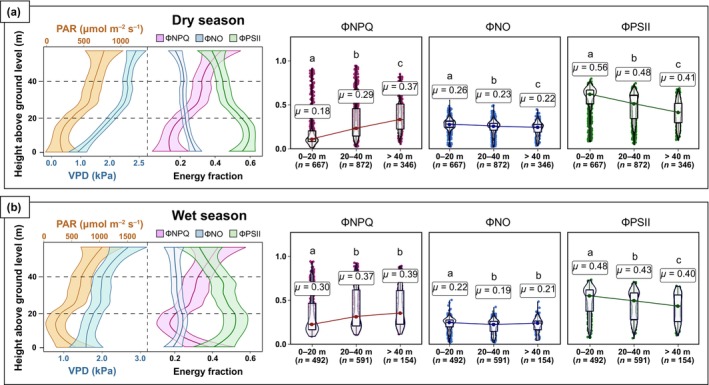
Left panels show confidence intervals (90%) for the vertical canopy profiles of microenvironmental variables (photosynthetic active radiation (PAR) and vapor pressure deficit (VPD)) and the yields for energy dissipative processes (ΦNO, nonregulated quenching including fluorescence re‐emission; ΦNPQ, heat dissipation; ΦPSII, photochemistry) sampled with the MultispeQ during dry (a) and wet (b) seasons. The right panels present boxplots and the average values (*μ*) for ΦPSII, ΦNO, and ΦNPQ in each canopy stratum. Boxplots include the median (represented by colored dots), the interquartile range for the first (25^th^) and third (75^th^) percentiles (shown as white boxes), data distribution (illustrated by white curves), and the minimum and maximum values (depicted as whiskers). Significant differences (Kruskal–Wallis test and Mann–Whitney *U* test) between groups are indicated by different lowercase letters (a–c).

The fate of absorbed photons varied with vertical canopy environments and between wet and dry seasons in our central Amazonian forest (Fig. 2). In general, both seasons exhibited similar patterns of microenvironmental variation, with PAR and VPD increasing with increasing height as expected. In response, ΦNPQ increased toward the upper canopy, while ΦPSII and ΦNO decreased. However, we found that these vertical quantum yield patterns were nonlinear, with inflections in the lower canopy and understory (S1). Small variations in ΦNO along the vertical forest profile corresponded to substantial compensatory changes in ΦNPQ and ΦPSII, and no significant difference in ΦNO was observed between the middle and upper canopy strata during the wet season. Intracanopy variations in photochemical and nonphotochemical yields were more pronounced in the dry season, with ΦNPQ increasing by 19%, while ΦPSII and ΦNO decreased by 15% and 4%, respectively, from the lowest to the upper canopy strata (Fig. 2).

We applied GMMs (Eqn 5) to examine the specific effects of environmental variables – specifically PAR, VPD, and height above the ground – and interspecific variation on photochemical and nonphotochemical quantum yields during dry and wet seasons at the TNF site. Consistent patterns were observed across all models, highlighting that microenvironmental drivers had a stronger influence than species variation on ΦNPQ, ΦNO, and ΦPSII. With the exception of the effect of height on ΦNO in the dry season, all microenvironmental predictors demonstrated significant impacts on the variation of the three quantum yields. The marginal *R* ^2^ (environmental effect) for the model using ΦNO as the response variable during the wet season was 0.53, which is 19% higher than the marginal *R* ^2^ value in the dry season (0.34) (Fig. S1), suggesting that stress decouples microenvironmental responses. For ΦNPQ, the difference in marginal *R* ^2^ values between the wet (0.79) and dry seasons (0.77) was only 2%, while for ΦPSII, the marginal *R* ^2^ was the same (0.81) for both seasons.

**Fig. 3:**
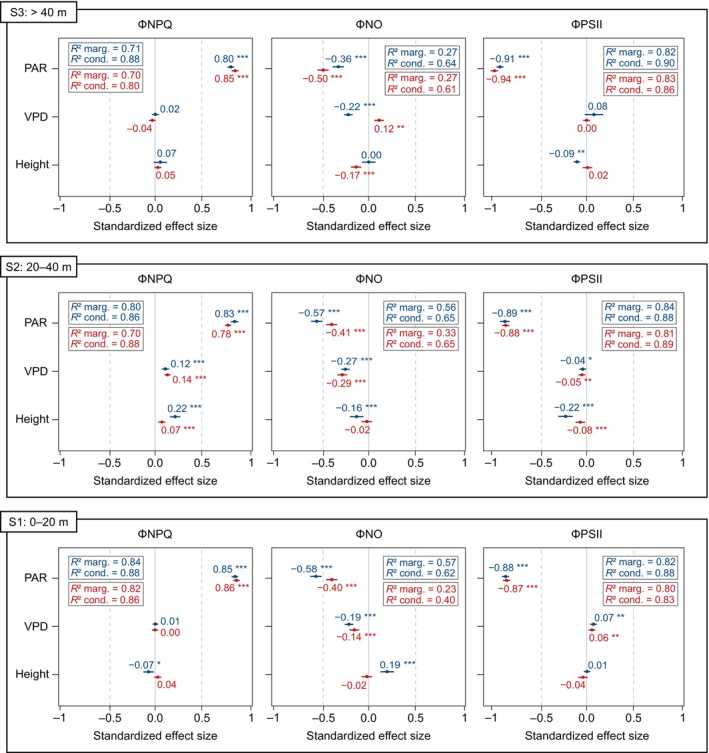
Standardized coefficients of each predictor variable in the generalized mixed models developed for each quantum yield (ΦNO, fluorescence re‐emission; ΦNPQ, heat dissipation; ΦPSII, photochemistry) across canopy strata (*, *P* < 0.01; **, *P* < 0.05; ***, *P* < 0.001). Blue and red colors represent wet and dry seasons, respectively. The boxes show marginal and conditional *R* ^2^ values, with species included as a random effect.

Considering the influence of environmental variables on the three quantum yields within canopy layers, PAR had the strongest effect, followed by VPD and height (Fig. 3). In the lower (S1) and mid‐canopy (S2), PAR had similar effects on ΦNPQ and ΦPSII across both seasons, while its impact on ΦNO was greater during the wet season. In the upper canopy (S3), PAR showed a stronger effect on all quantum yields during the dry season, whereas VPD did not significantly affect ΦNPQ and ΦPSII within this stratum. Furthermore, the effect of VPD on ΦNO transitions from being negative to positive as canopy height increases during the dry season. In general, height had more pronounced effects on the three quantum yields in S2, particularly during the wet season. Significant height effects were also observed for ΦNPQ and ΦNO in S1 during the wet season, for ΦPSII in S3 during the wet season, and for ΦNO in S3 during the dry season.

**Fig. 4:**
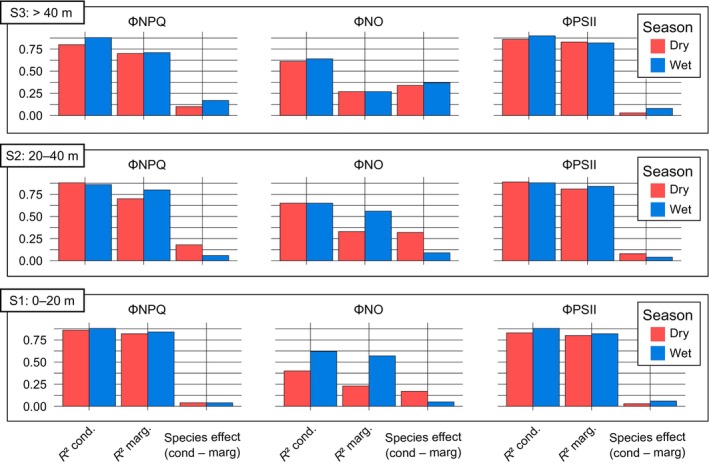
Conditional and marginal *R* ^2^ values from the generalized mixed models developed for each quantum yield (ΦNO, fluorescence re‐emission; ΦNPQ, heat dissipation; ΦPSII, photochemistry) across canopy strata. Dry and wet seasons are represented by red and blue bars, respectively. The species effect is represented by the difference between conditional and marginal *R* ^2^ values (Supporting Information Table S3).

The species (random) effect, given by the difference between conditional and marginal *R* ^2^, was more pronounced for ΦNO, particularly during the dry season, while it had minimal impact on ΦNPQ and ΦPSII. The interspecific variation accounted for nearly half (40%) of the conditional *R* ^2^ for ΦNO in both seasons in the upper canopy (S3) and during the dry season in the mid‐canopy (S2). These findings provide strong evidence that SIF may be highly influenced by interspecific variation within tropical forest canopies (Fig. 4).

**Fig. 5:**
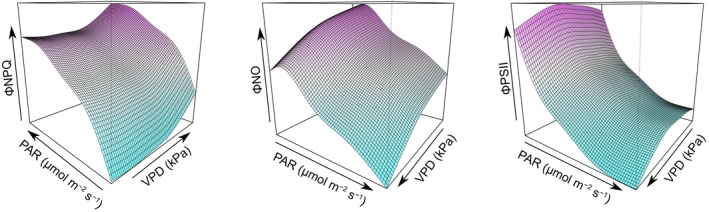
Generalized additive mixed models of ΦNPQ, ΦNO, and ΦPSII, as functions of photosynthetic active radiation (PAR) and vapor pressure deficit (VPD), and height included as predictors, and species as a random effect. The plots show 3D visualizations of the interaction effects between VPD and PAR on the quantum yields, where the color gradients show low (green) to high (pink) quantum yield values.

Our GAMMs, with smoothing terms adjusted by season (Eqn 6), revealed distinct patterns of variation in the three quantum yields in relation to PAR and VPD. ΦNPQ exhibited a positive logarithmic increase with PAR and an exponential increase with VPD at the whole‐forest level (Fig. 5). By contrast, both PAR and VPD negatively affected ΦNO and ΦPSII. We observed a stronger effect of VPD on ΦNO in comparison with the other quantum yields. These GAMMs provided an integrated analysis of the partial effects of PAR and VPD, confirming the patterns observed in the GMMs. The hierarchical partitioning of explained deviance in our models indicates that PAR was the primary driver of variation in ΦPSII and ΦNPQ (Table S4). However, VPD and interspecific variation together accounted for *c*. 43% of the total deviance explained in the model for ΦNO, highlighting their significant roles in shaping leaf fluorescence dynamics. ΦPSII showed an exponential decrease to increasing PAR, while ΦNPQ followed a logarithmic increase. ΦNO values were relatively stable under low‐to‐moderate PAR before gradually declining at higher light intensities and VPD levels.

**Fig. 6:**
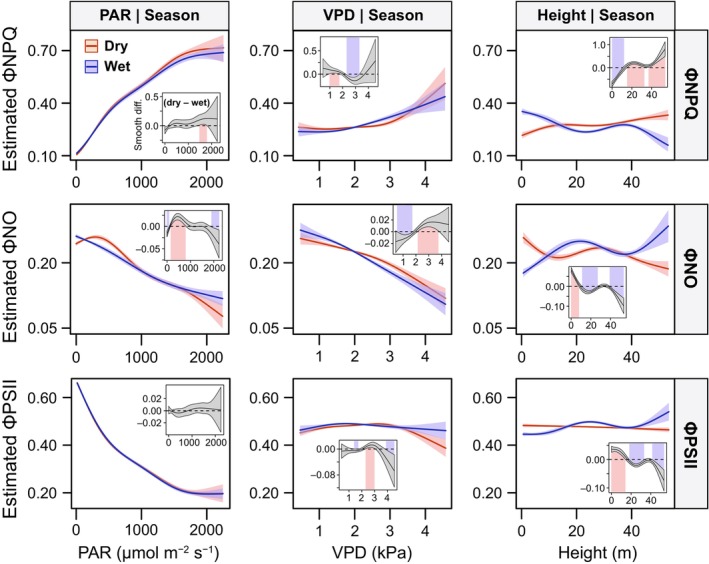
Estimated smooth effects of each predictor in the generalized additive mixed models for the dry and wet seasons (red and blue lines, respectively), with 95% confidence intervals represented by the corresponding shaded areas. The small inset panels within each plot show seasonal differences in curves adjusted for photosynthetic active radiation (PAR) and vapor pressure deficit (VPD), and height. The solid black line represents the estimated difference in smoothing terms between seasons (dry − wet), with the 95% confidence interval shown by the grey shaded area. The dashed horizontal black line at zero indicates no difference between seasons. Significant seasonal differences occur in which the confidence interval of the differences does not overlap zero. These areas are highlighted with the vertical red and blue bars to indicate significantly higher values in the dry and wet seasons, respectively.

We observed significant seasonal differences in the smooth effects of PAR, VPD, and height on the three quantum yields. The relative stability of ΦNO under low‐to‐moderate PAR levels was driven by contrasting seasonal patterns. In the dry season, PAR had a positive effect on ΦNO up to *c*. 300 μmol m^−2^ s^−1^, and a negative effect at higher light intensities (Fig. 6). By contrast, during the wet season, ΦNO steadily decreased as PAR increased. As a result, ΦNO was significantly higher in the dry season across a broad PAR range, up to *c*. 900 μmol m^−2^ s^−1^. However, no seasonal differences were observed for the effects of PAR on ΦPSII, and a small increase in ΦNPQ was observed over a narrow PAR range under high light during the dry season. At intermediate VPD values (2–3 kPa), ΦNO was significantly higher in the dry season, while ΦNPQ was significantly lower. As VPD increased above 4 kPa, there were no significant seasonal changes in ΦNO or ΦNPQ, although ΦPSII was significantly higher in the wet season. Our GAMMs also revealed intracanopy seasonal shifts in the quantum yields along the vertical profile. In the lower canopy (< 15 m), ΦPSII and ΦNO were significantly higher in the dry season, accompanied by lower ΦNPQ. By contrast, in the middle and upper canopy strata, higher ΦPSII and ΦNO, along with lower ΦNPQ, were observed in the wet season. These findings highlight how canopy environments affect physiological adjustments of leaf energy dissipation and photosynthetic processes in different forest strata, which is key for understanding leaf‐level quantum yield dynamics under environmental stress and accurately interpreting tower and satellite SIF signals.

**Fig. 7:**
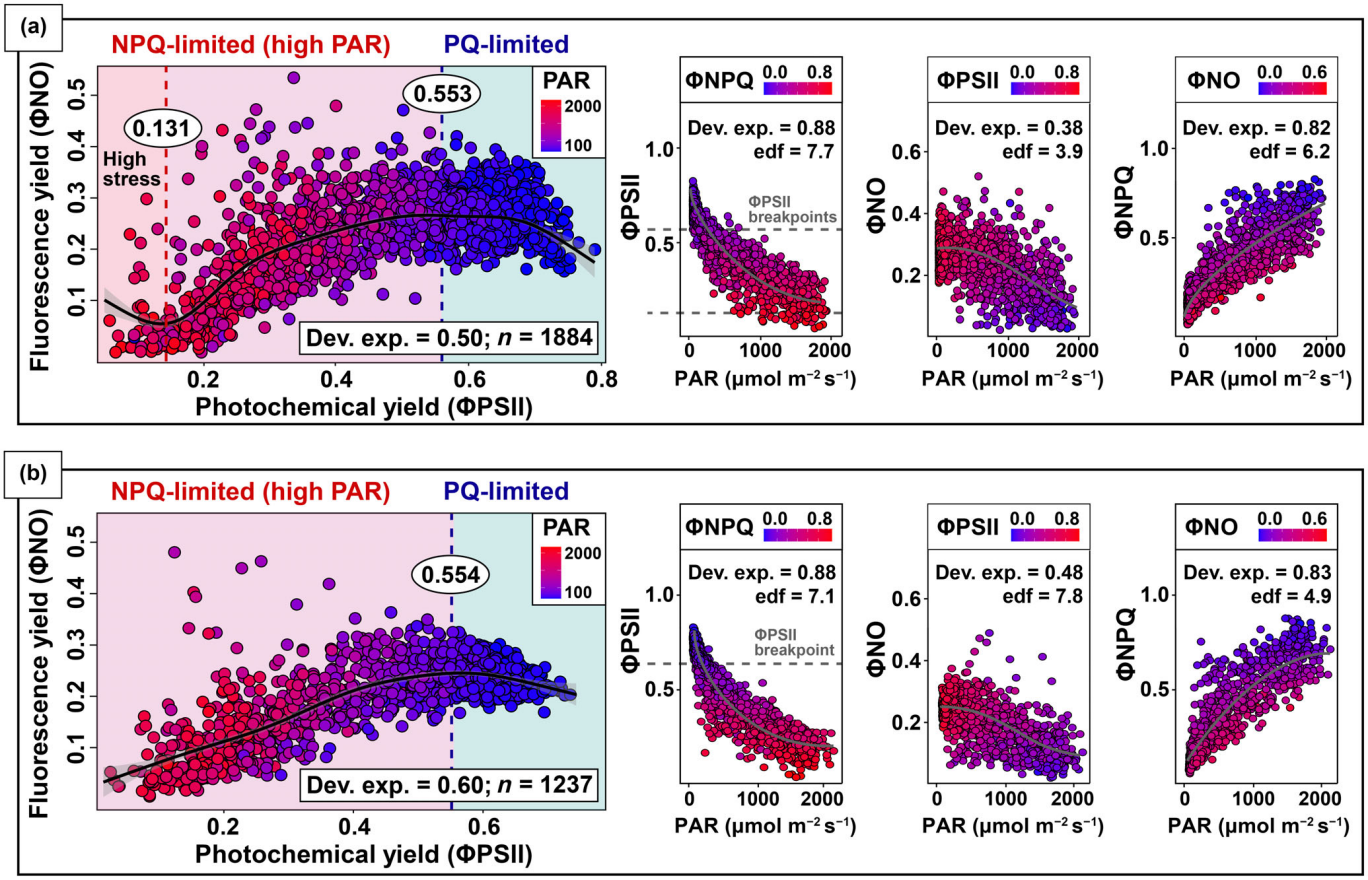
Generalized additive models (GAMs) fitted to steady‐state fluorescence yield (ΦNO) and photosystem II yield (ΦPSII), also showing the deviance explained (Dev. exp.) and the effective degrees of freedom (edf) of GAMs fitted for the relationships between each of the three quantum yields (ΦNO, fluorescence re‐emission; ΦNPQ, heat dissipation; ΦPSII, photochemistry) and photosynthetic active radiation (PAR; in μmol m^−2^ s^−1^). Grey shaded areas represent 95% confidence intervals. Panels (a, b) correspond to observations from the dry and wet seasons, respectively.

The seasonal variation in the relationship between ΦPSII and ΦNO was examined using GAMs fitted separately for the dry (deviance explained = 0.50) and wet (deviance explained = 0.60) seasons. Two breakpoints were detected in the relationship during the dry season, whereas a single inflection point associated with a phase shift was observed during the wet season (Fig. S2). In the dry season, the ΦPSII breakpoints were identified at 0.131 and 0.553. The PAR values associated with these breakpoints were estimated by our GAMs as 1977.1 (±102.9) and 151.1 (±7.9) μmol m^−2^ s^−1^, respectively. The latter breakpoint marked a transition from a phase characterized by photochemical quenching limitation (PQ‐limited) under low PAR to a phase dominated by nonphotochemical quenching limitation (NPQ‐limited) under high PAR. The ΦPSII breakpoint associated with this phase shift for the wet season was 0.554, a similar value to that observed in the dry season. The PAR value associated with this breakpoint was estimated as 150.2 (±9.9) μmol m^−2^ s^−1^. Relationships between each one of the three quantum yields and PAR were consistent between seasons. Our seasonal GAMs also revealed nonlinear relationships (edf > 1) between each of the three quantum yields (ΦNO, ΦPSII, and ΦNPQ) and PAR for both the dry and wet seasons. In the dry season, the deviance explained values indicate a higher goodness of fit for ΦPSII (0.88), followed by ΦNPQ (0.82), and ΦNO (0.38). Similarly, in the wet season, the deviance explained values indicate a comparable pattern with values of 0.88, 0.83, and 0.48 for ΦPSII, ΦNPQ, and ΦNO, respectively (Fig. 7).

The observed patterns between each of the quantum yields and PAR were consistent across all canopy strata. Specifically, the relationship between ΦPSII and PAR demonstrated a negative exponential decay, whereas both ΦNO and ΦNPQ showed nonlinear positive relationships with PAR across the entire canopy profile (Fig. S3). We also observed that the adjusted *R* ^2^ values of the ΦNPQ‐PAR relationship decreased as height increased in both seasons. In the lower canopy stratum (S1), this relationship followed a more pronounced exponential pattern, indicating ΦNPQ saturation in lower canopy trees as PAR levels increased, a pattern not observed in the middle and upper canopy strata in which trees are adapted to higher light regimes.

We found significant increases in NPQt and decreases in qL from the lower to the upper canopy, highlighting intracanopy physiological adjustments that regulate variations in photochemical and nonphotochemical yields (Fig. S4). However, during the wet season, no significant differences were observed in NPQt between S2 and S3, and in qL, between S1 and S2. PAR showed strong nonlinear positive and negative relationships with NPQt and qL, respectively (Fig. S5; Table S5). VPD also showed significant relationships with both variables across all canopy strata and seasons, except for NPQt in S1 during the dry season, and qL in S2 in both seasons. Linear relationships were observed between VPD and both NPQt and qL in S3 during the dry season, as well as between VPD and qL in S1 and S3 during the wet season (Table S5).

## Discussion

The partitioning of energy into competing photochemical and nonphotochemical pathways varied significantly along the vertical forest profile, revealing the importance of canopy environments to leaf energy partitioning and the emergent SIF signal observed above the canopy (e.g. from satellite remote sensing). We found significantly higher ΦNO (the signal underlying SIF) associated with lower PAR and VPD values in the lower canopy. While PAR was the main environmental driver for the variation of all three quantum yields across canopy strata and seasons, we also found important interspecific effects associated with variations in ΦNO, especially in the upper canopy. The overall relationship between ΦNO and the photosynthetic yield, ΦPSII, was strongly nonlinear, with a broad region under low PAR in which variations in the photochemical yield were negatively related to fluorescence. A negative relationship was also observed under high PAR during the dry season, implying that SIF cannot consistently predict GPP under low‐light conditions or when energy dissipation via NPQ is high in response to excessive irradiation and likely coupled to reduced soil water availability.

When light is low and NPQ energy dissipation is minimal, there is a negative linear trade‐off between fluorescence and photochemistry (‘PQ phase’). As light increases, NPQ dissipation dominates the fates of absorbed energy, inflecting the relationship to positive covariation between ΦNO and ΦPSII (‘NPQ phase’). However, in a ‘high stress phase’, high NPQ heat dissipation may damage PSII reaction centers and cause photoinhibition, again driving a negative relationship (Porcar‐Castell *et al*., 2014). Identifying this phase provides important insights into environmental impacts on photosynthetic function (Magney *et al*., 2020). Furthermore, this high stress phase may cause erroneous interpretation of satellite SIF observations during drought and heat stress particularly (Magney *et al*., 2020; Porcar‐Castell *et al*., 2021; Martini *et al*., 2022). Failure to incorporate this multiphasic leaf‐level physiology in estimation of ecosystem photosynthetic responses could result in reduced capacity of pan tropical data sources to assess climate sensitivities, while, conversely, with proper characterization, this knowledge could help reveal important canopy function tipping points.

Similar patterns of intracanopy microenvironmental variability were observed in both seasons, characterized by increases in PAR and VPD with increasing canopy height. These patterns mirrored intracanopy variations in photochemical and nonphotochemical yields, in which ΦNPQ increased while ΦPSII and ΦNO decreased from the lower to the upper canopy stratum (Fig. 2). Given the high leaf area density in the lower canopy of central Amazon forests (Stark *et al*., 2012; Smith *et al*., 2019), and the higher fluorescence yield associated with lower NPQ in shaded leaves under low VPD levels as shown here, a large fraction of the overall SIF measured from above‐canopy sensors is expected to originate from this stratum. However, more research will be needed to assess this expectation.

The relationship between ΦPSII and PAR followed a negative exponential decay, while ΦNO and ΦNPQ exhibited nonlinear negative and positive relationships with PAR, respectively. These patterns corroborate previous studies focused on the relationship between these quantum yields and light availability (Hendrickson *et al*., 2004; Kramer *et al*., 2004; Klughammer & Schreiber, 2008; Scartazza *et al*., 2016). Variations in ΦNO values across the vertical forest profile were observed within a smaller range than variations in ΦPSII and ΦNPQ, suggesting a relative stability of leaf‐level SIF over canopy environments, with major compensatory changes in photochemical and NPQ. While emergent whole‐canopy variations in ΦNO may be tracked by tower and satellite SIF observations, the intracanopy compensatory changes in ΦPSII and ΦNPQ linked to canopy environments cannot, adding complexity to instantaneous GPP inferences from SIF in structurally complex and diverse tropical ecosystems. In this context, our findings reveal seasonal and subcanopy fractions of steady‐state quantum yields *in vivo* that affect the overall SIF signal in tropical forests, while shedding light on nonlinearities in the relationship between ΦNO and ΦPSII.

As observed by Castro *et al*. (2020), the canopy SIF signal in Amazonian forests – where irradiation is a key driver of tree species distribution (Lima *et al*., 2023) – may also be influenced by species composition. Steady‐state ChlF is species‐dependent (Björkman & Demmig, 1987) and also affected by shading (Khan *et al*., 2000; Mauro *et al*., 2011), leaf age (Bielczynski *et al*., 2017), and nitrogen availability (Lin *et al*., 2013). Our integrated analysis using mixed and additive multiple models indicates that although PAR had the most significant effect on the variation of all three quantum yields among the microenvironmental predictors (Fig. 3), VPD and interspecific effects also accounted for a large part of ΦNO variation (Table S4), particularly in the upper and middle canopy strata, and during the dry season (Fig. 4). In spite of these species‐specific effects, our results confirm that PAR is a major environmental driver of variations in the yields for energy dissipative processes in tropical forests, similar to Wyber *et al*. (2017), who identified leaf irradiance as the primary predictor of SIF at the canopy scale for two C_3_ plant species in Australia. Pierrat *et al*. (2022) also found that PAR is the most important predictor for short‐term (i.e. half‐hourly) models of SIF and GPP predictions in boreal ecosystems, although additional temperature‐related factors regulate these variables on a seasonal scale.

While most of the SIF variation in temperate forests is explained by the fraction of total radiance absorbed by vegetation (fPAR) and APAR (Li *et al*., 2018a), the complexity of tropical forest canopies makes the interpretation of SIF challenging (Castro *et al*., 2020; Porcar‐Castell *et al*., 2021). In addition to irradiance, previous research in Amazonian forests emphasizes the roles of water availability and adaptive mechanisms linked to photosynthetic capacity (i.e. canopy phenology and litterfall seasonality) in regulating ecosystem productivity (Restrepo‐Coupe *et al*., 2013; Wu *et al*., 2016, 2017). Our GAMMs indicate that VPD also plays a crucial role in regulating ΦNO at the leaf level, and consequently, in driving SIF variation across seasons in tropical forest ecosystems (Figs 3, 5, 6). The elevated ΦNO values observed across all canopy strata during the dry season, along with the increased effect of VPD on ΦNO in the wet season, are the first field‐based leaf‐scale evidence that align with previous satellite SIF observations of SIF increases during the dry season (Doughty *et al*., 2019, 2021b) and higher sensitivity of SIF to VPD during the wet season in Amazonian forests (Green *et al*., 2020).

Gu *et al*. (2019) demonstrated how the dynamics of NPQ and qL – regulated heat dissipation and the fraction of open PSII centers, respectively – affect the sensitivity of GPP to SIF. The relatively low sensitivity of leaf‐level fluorescence to light variation is due to compensating effects of NPQ and qL, which tend to shift in opposite directions as PAR increases (Fig. S5). As a result, the joint dynamics of NPQ and qL have a limited capacity to regulate SIF responses to light, leading to a continuous increase in SIF with increasing PAR, while GPP saturates due to carboxylation limitations in C_3_ species. These limitations stem from CO_2_ partial pressure in the stroma of chloroplasts, constrained by stomatal and mesophyll diffusion. According to the fundamental equation describing the relationship between actual electron transport rate (*J*) and GPP, qL dynamics also dominate variations in ΦPSII, thereby modulating *J* responses to PAR variations and consequently, GPP (Gu *et al*., 2019). In this context, we found intracanopy variations in NPQ and qL across seasons (Fig. S4), along with significant relationships between these variables with PAR and VPD (Fig. S5). While developing a universal leaf‐level mechanistic model linking SIF and photosynthesis across canopy strata and environmental conditions remains an essential next step for upscaling analyses, our insights can help to refine SIF‐based models of photosynthesis in structurally complex tropical forests (e.g. Z. Liu *et al*., 2024).

Our study also provides the first tropical forest evidence of a nonlinear relationship between ΦNO and ΦPSII across both seasons (Fig. 7). This result corroborates the theory and previous experimental studies that revealed a positive‐to‐negative inversion in the proportionality of these quantum yields (Porcar‐Castell *et al*., 2014; Van der Tol *et al*., 2014; Maguire *et al*., 2020; Pierrat *et al*., 2024), and others that show a nonlinear relationship between leaf‐level fluorescence and carbon assimilation (Magney *et al*., 2017; Frankenberg & Berry, 2018; Gu *et al*., 2019). The PQ‐limited to NPQ‐limited transition breakpoints of the relationship between ΦNO and ΦPSII of both dry and wet seasons (0.553 and 0.554, respectively) were lower than values previously reported in temperate forests (ΦPSII = 0.6–0.76) (Maguire *et al*., 2020). These lower breakpoints may reflect the adaptation of trees in Amazonian forests to sustained high irradiance levels.

While a recent study found a high correlation between GPP from three eddy flux sites in Amazonian forests and OCO‐3 SIF modeled using a machine learning approach in 1‐h intervals each month from 2015 to 2021 (Zhang *et al*., 2023), validation of global scale‐positive linear relationships between GPP and SIF has significantly lagged in tropical forests where satellite data are limited and canopy‐scale SIF and GPP data are lacking (Li *et al*., 2018b; Sun *et al*., 2018). Furthermore, the increase in SIF during the dry season in tropical forest ecosystems can be attributed to factors such as an increase in the Chl content within the canopy, the shedding of old leaves, and the flushing of new leaves (Doughty *et al*., 2019). These phenomena have been documented through various methods, including *in situ* litterfall traps (Rice *et al*., 2004), tower‐based time‐lapse photography (Lopes *et al*., 2016), and satellite‐derived vegetation indices (Xiao *et al*., 2006). While this study provides insights into seasonal leaf energy partitioning along the vertical forest profile, which may enhance future GPP predictions from SIF, our dataset was collected exclusively from mature leaves. Further studies in complex tropical forest canopies should consider the effects of leaf age on energy dissipation and photosynthesis at multiple heights, unifying vertical and interspecific variations with microenvironmental conditions into a cohesive mechanistic model.

Solar‐induced Chl fluorescence observations from spaceborne sensors are related to emitted, and not reflected energy, which avoids artifacts related to vegetation reflectance indices. However, although SIF is affected by cloud cover to a smaller degree than surface reflectance data (Guanter *et al*., 2015; Köhler *et al*., 2018a; Doughty *et al*., 2021b), cloudy sky conditions are a common feature of Amazonian forests, and the availability of satellite‐based retrievals of SIF for this biome is restricted to sunny sky and high‐light conditions. Due to the inability to monitor SIF under cloudy conditions from space, GPP inferences from satellite SIF observations may be biased in relationships derived from cloud‐free data (NPQ‐limited) that may be extrapolated over and applied to cloudy days (PQ‐limited) (Zhao *et al*., 2005; Ryu *et al*., 2019). Parazoo *et al*. (2014) highlight that the effects of diffuse light and photochemical responses to light saturation are primary sources of bias associated with satellite SIF observations. In this context, our study shows the critical role of canopy environments and points to a large contribution of lower canopy leaves to the overall SIF signal in tropical forests. These insights combined with information about forest structure (i.e. leaf area density profiles) can be used to enhance the accuracy of GPP predictions derived from SIF models, especially under cloudy sky conditions.

By providing insights into season‐specific microenvironmental and quantum dynamics, this study contributes to enhancing predictions of forest photosynthesis and adaptability to environmental changes. We have demonstrated within‐canopy and seasonal variations in the fluxes of absorbed photons through photochemical (ΦPSII) and nonphotochemical (ΦNO and ΦNPQ) pathways. Leveraging a novel and unique dataset collected at multiple canopy heights for the most abundant tree species in central Amazonia, our results revealed the first tropical forest evidence of a nonlinear relationship between ΦNO and ΦPSII, highlighting the nonlinear nature of the relationships between SIF yield and GPP at the canopy level. This can lead to erroneous interpretation of satellite SIF observations, especially during dry seasons, in which a triphasic relationship between ΦNO and ΦPSII was observed and leaf‐level SIF‐GPP reversals due to stress may alter the ecosystem‐level relationship. Furthermore, since a large fraction of above‐canopy SIF in structurally complex tropical canopies may originate not only from sunlit leaves in the top of the canopy but also from shaded leaves, we highlight the importance of accounting for subcanopy fractions of PQ‐ and NPQ‐limited foliage for accurate assessments of SIF‐GPP relationships in tropical forests.

Our findings suggest that including intracanopy variations in leaf quantum yields in SIF analyses may improve predictions of GPP for terrestrial biosphere monitoring and modeling. The implications presented here will facilitate the effective use of high‐frequency observations from tower and satellite‐based sensors in studies concerning plant functioning and ecosystem processes, ultimately enhancing our ability to forecast future changes in terrestrial carbon and water cycles.

## Competing interests

DK is a cofounder of the open science PhotosynQ and MultispeQ platforms used in the study.

## Author contributions

LGZ, DK, NG, TT and SCS conceived the ideas and designed methodology. LGZ, NP and KSC collected the data. LGZ, NG, BWN, LPA, NR‐C, SRS and DK analyzed and interpreted the data. LGZ and SCS led the writing of the manuscript. All authors contributed critically to the drafts and gave final approval for publication.

## Disclaimer

The New Phytologist Foundation remains neutral with regard to jurisdictional claims in maps and in any institutional affiliations.

## Supporting information


**Fig. S1** Standardized coefficients of each predictor variable in the generalized mixed models developed for each quantum yield.
**Fig. S2** Confidence intervals (95%) of the derivatives of the generalized additive models.
**Fig. S3** Generalized additive models with 95% confidence intervals fitted to the three quantum yields (ΦNO, ΦPSII, and ΦNPQ) and photosynthetic active radiation in each canopy stratum.
**Fig. S4** Boxplot and the average values for NPQt and qL in each canopy stratum and season.
**Fig. S5** Generalized additive models with 95% confidence intervals showing the relationships between NPQt and qL (responses) with photosynthetic active radiation and vapor pressure deficit (predictors) across seasons and canopy strata.
**Methods S1** Generalized additive models fitted to the relationships between qL and NPQt with photosynthetic active radiation and vapor pressure deficit.
**Table S1** Scientific names of the sampled tree species at the K67 site.
**Table S2** Variance inflation factor for the predictors of generalized mixed models developed for each quantum yield.
**Table S3** Conditional and marginal *R* ^2^ values from the generalized mixed models developed for each quantum yield.
**Table S4** Hierarchical partitioning of explained deviance for generalized additive mixed models.
**Table S5** Outputs of generalized additive model smooth terms for the relationships between NPQt and qL (responses) with photosynthetic active radiation and vapor pressure deficit (predictors) across seasons and canopy strata.
Please note: Wiley is not responsible for the content or functionality of any Supporting Information supplied by the authors. Any queries (other than missing material) should be directed to the *New Phytologist* Central Office.

## Data Availability

Data for this study are freely available at doi: 10.5061/dryad.tb2rbp0cr.
