# Supplementary material for: Seasonal and intracanopy shifts in the fates of absorbed photons in central Amazonian forests: implications for leaf fluorescence and photosynthesis
Source: New Phytol. 2025 May 29;248(1):76–91. doi: 10.1111/nph.70183 (PMC12409102; doi:10.1111/nph.70183)
Supplement: Supplementary file 1 — Fig. S1 Standardized coefficients of each predictor variable in the generalized mixed models developed for each quantum yield. Fig. S2 Confidence intervals (95%) of the derivatives of the generalized additive models. Fig. S3 Generalized additive models with 95% confidence intervals fitted to the three quantum yields (ΦNO, ΦPSII, and ΦNPQ) and photosynthetic active radiation in each canopy stratum. Fig. S4 Boxplot and the average values for NPQt and qL in each canopy stratum and season. Fig. S5 Generalized additive models with 95% confidence intervals showing the relationships between NPQt and qL (responses) with photosynthetic active radiation and vapor pressure deficit (predictors) across seasons and canopy strata. Methods S1 Generalized additive models fitted to the relationships between qL and NPQt with photosynthetic active radiation and vapor pressure deficit. Table S1 Scientific names of the sampled tree species at the K67 site. Table S2 Variance inflation factor for the predictors of generalized mixed models developed for each quantum yield. Table S3 Conditional and marginal R 2 values from the generalized mixed models developed for each quantum yield. Table S4 Hierarchical partitioning of explained deviance for generalized additive mixed models. Table S5 Outputs of generalized additive model smooth terms for the relationships between NPQt and qL (responses) with photosynthetic active radiation and vapor pressure deficit (predictors) across seasons and canopy strata. Please note: Wiley is not responsible for the content or functionality of any Supporting Information supplied by the authors. Any queries (other than missing material) should be directed to the New Phytologist Central Office. [file NPH-248-76-s001.docx]

## *New Phytologist* Supporting Information

Article title: Seasonal and intra-canopy shifts in the fates of absorbed photons in central Amazonian forests: Implications for leaf fluorescence and photosynthesis

Authors: Leonardo G. Ziccardi, David Kramer, Nathan Gonçalves, Bruce W. Nelson, Tyeen Taylor, Loren P. Albert, Kleber S. Campos, Neill Prohaska, Natalia Restrepo-Coupe, Scott R. Saleska, Scott C. Stark

Article acceptance date: 7 April 2025

The following Supporting Information is available for this article:

**Fig. S1** Standardized coefficients of each predictor variable in the generalized mixed models developed for each quantum yield.

**Fig. S2** Confidence intervals (95%) of the derivatives of the generalized additive models (GAMs).

**Fig. S3** Generalized additive models (GAMs) with 95% confidence intervals fitted to the three quantum yields (ΦNO, ΦPSII, and ΦNPQ) and photosynthetic active radiation (PAR) in each canopy stratum.

**Fig. S4** Boxplot and the average values for NPQt and qL in each canopy stratum and season.

**Fig. S5** Generalized additive models (GAMs) with 95% confidence intervals showing the relationships between NPQt and qL (responses) with PAR and VPD (predictors) across seasons and canopy strata.

**Table S1** Scientific names of the sampled tree species at the K67 site.

**Table S2** Variance inflation factor (VIF) for the predictors of generalized mixed models (GMMs) developed for each quantum yield.

**Table S3** Conditional and marginal R² values from the Generalized Mixed Models (GMMs) developed for each quantum yield.

**Table S4** Hierarchical partitioning of explained deviance for Generalized Additive Mixed Models (GAMMs).

**Table S5** Outputs of generalized additive models (GAMs) smooth terms for the relationships between NPQt and qL (responses) with PAR and VPD (predictors) across seasons and canopy strata.

**Methods S1** Generalized additive models (GAMs) fitted to the relationships between qL and NPQt with PAR and VPD.

**Fig. S1** Standardized coefficients of each predictor variable in the generalized mixed models (GMMs) developed for each quantum yield (*p* < 0.05*; *p* < 0.001***). Blue and red colors represent wet and dry seasons, respectively. The boxes show marginal and conditional R² values, with species included as a random effect.


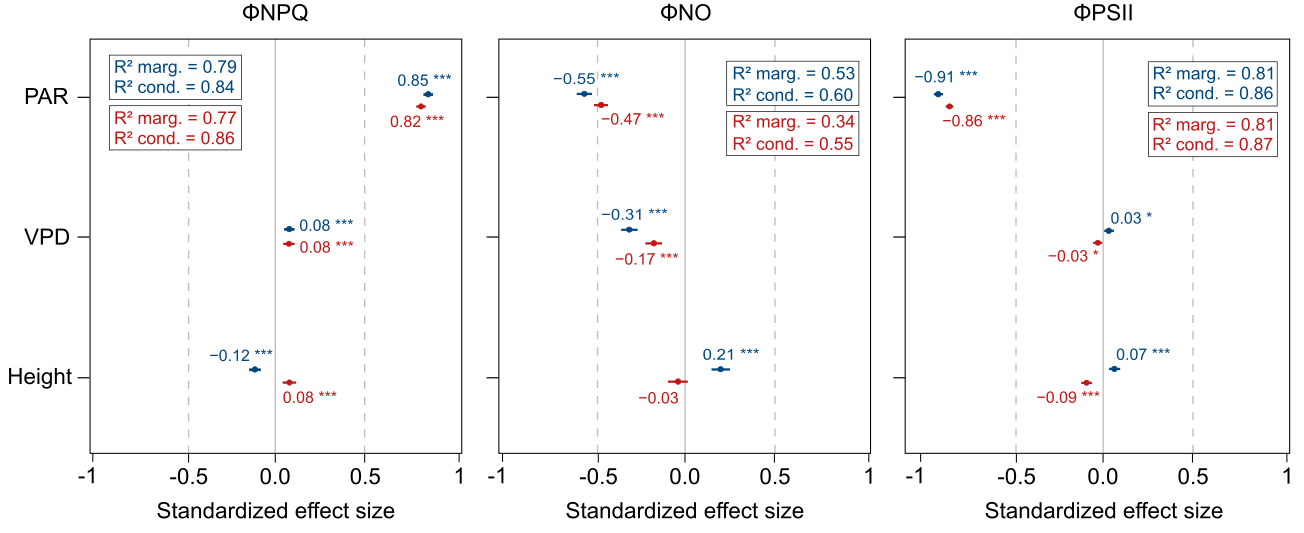


**Fig. S2** Confidence intervals (95%) of the derivatives of the generalized additive models (GAMs) for the relationship between photochemical yield (ΦPSII) and fluorescence yield (ΦNO). The inflection points, represented by red and blue dots and dashed lines, indicate the transition points where the derivatives transition from positive to negative. The blue breakpoints mark the transitions from a phase characterized by photochemical quenching limitation (PQ-limited) under low PAR to a phase dominated by non-photochemical quenching limitation (NPQ-limited) under high PAR. The red breakpoint marks the transition from the NPQ-limited phase to a high stress phase.


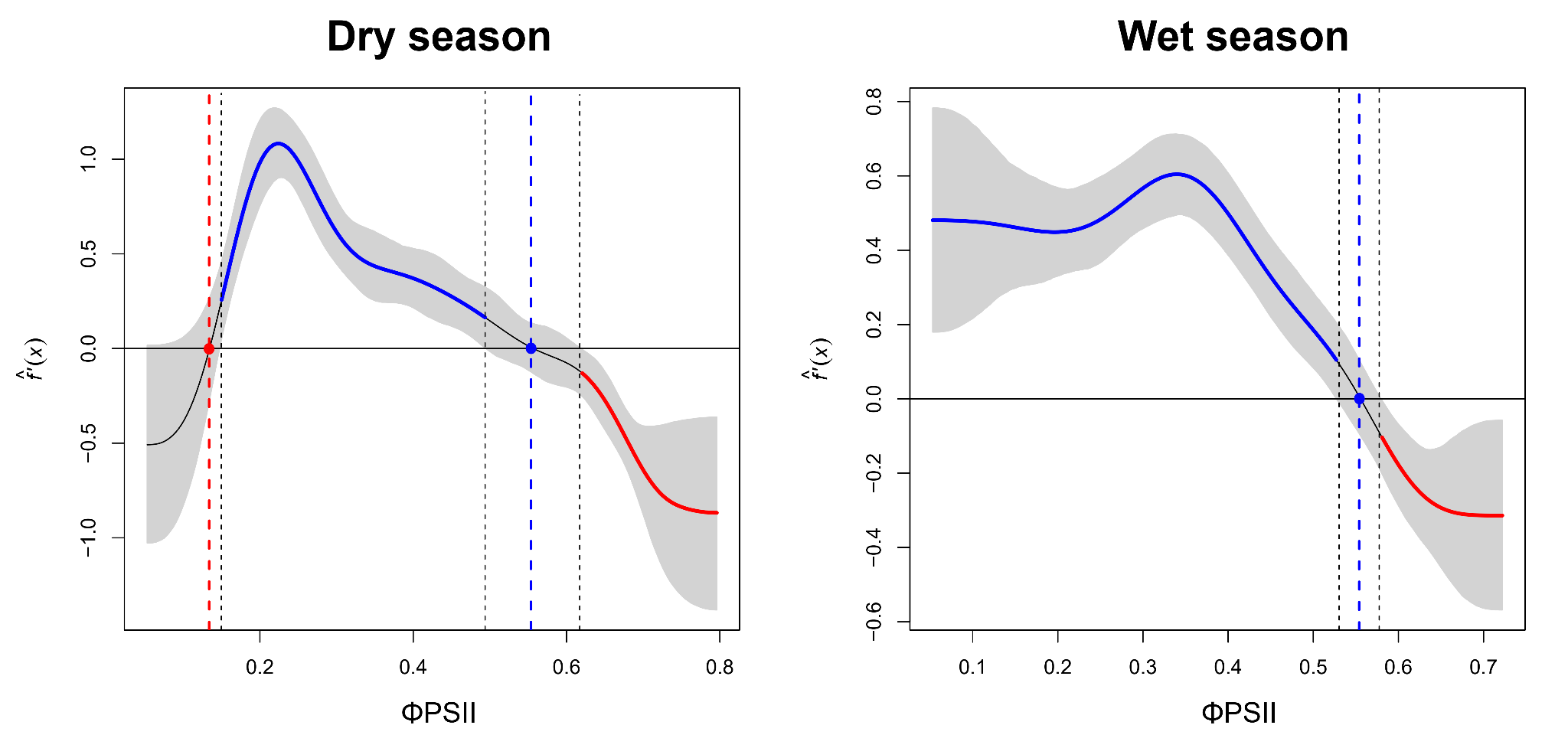


**Fig. S3** Generalized additive models (GAMs) with 95% confidence intervals fitted to the three quantum yields (ΦNO, ΦPSII, and ΦNPQ) and photosynthetic active radiation (PAR) in each canopy stratum (panels). The wet and dry seasons are represented in blue and red, respectively.


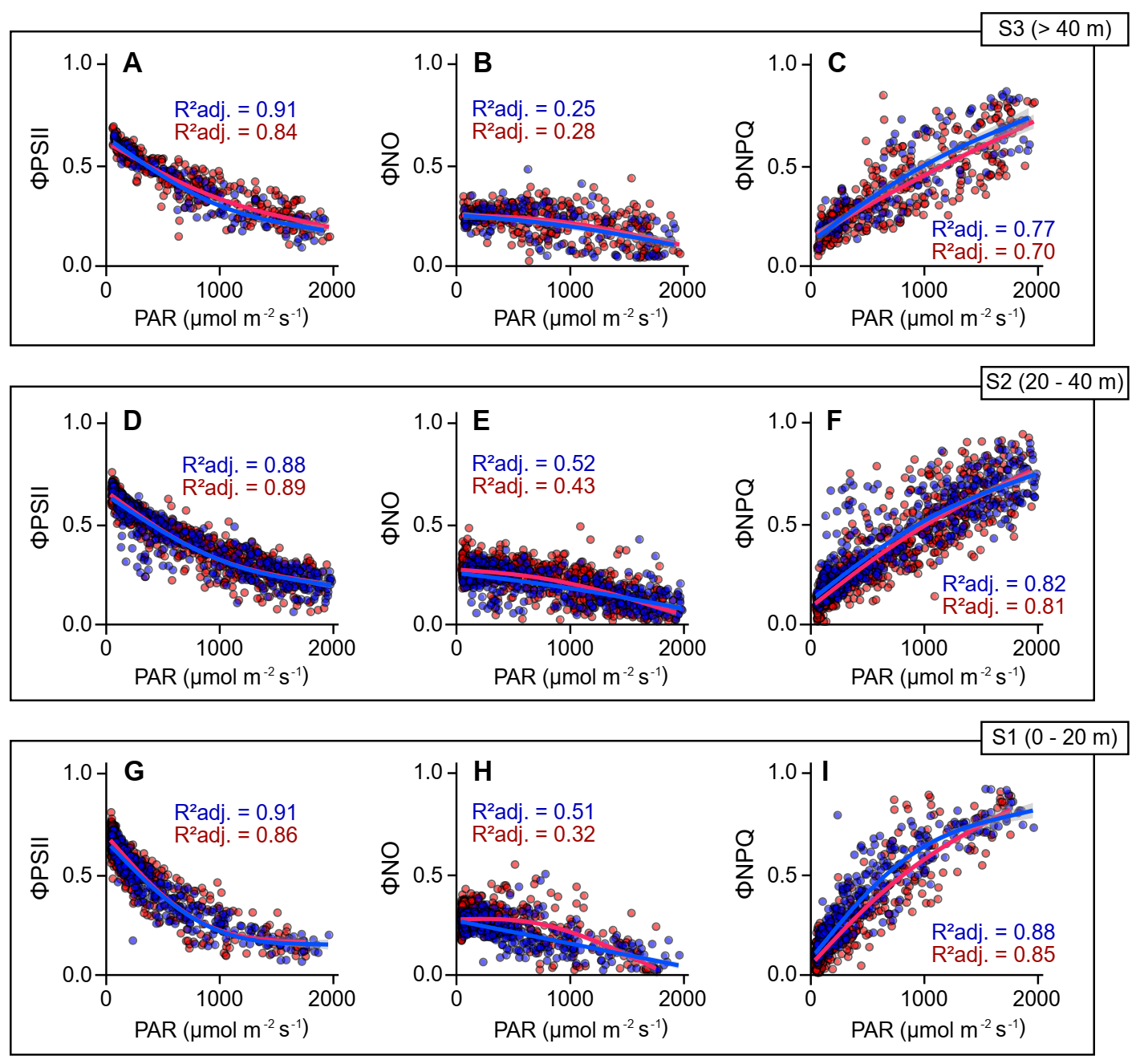


**Fig. S4** Boxplot and the average values (µ, black dots) for NPQt (blue) and qL (red) in each canopy stratum and season. Boxplots include the median, the interquartile range for the first (25th) and third (75th) percentiles (shown as boxes), data distribution (illustrated by curves), and the minimum and maximum values (depicted as whiskers). Significant differences (Kruskal-Wallis & Mann-Whitney U test) between groups are indicated by different lowercase letters (a, b, and c).


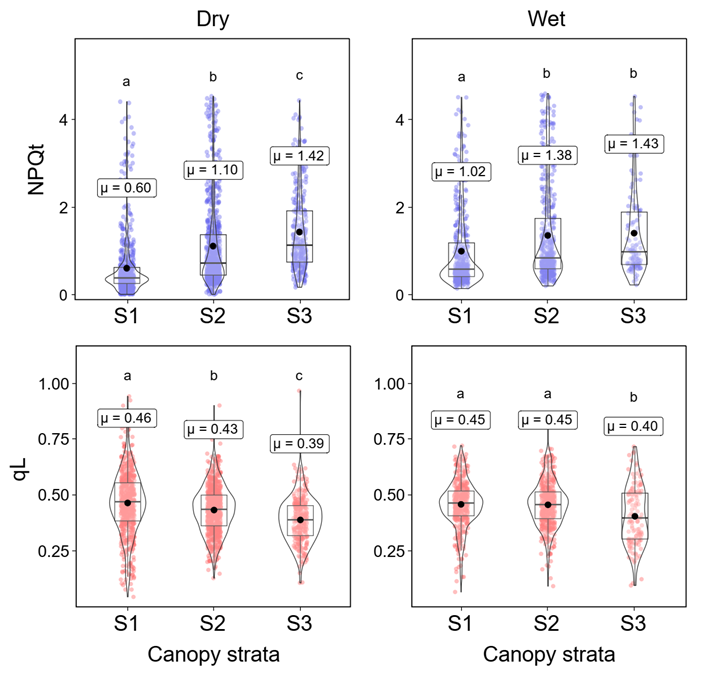


**Fig. S5** Generalized additive models (GAMs) with 95% confidence intervals showing the relationships between NPQt (blue) and qL (red) with PAR and VPD across seasons (panels a and b) and canopy strata (panels c and d). Model outputs are provided in Table S5.

**
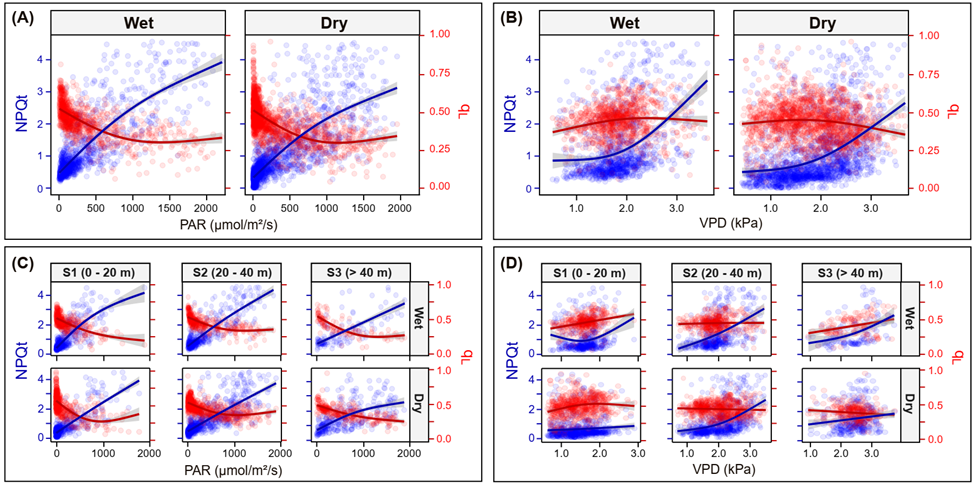
**

**Table S1** Scientific names of the sampled tree species in each canopy vertical strata at the K67 site.

| Season | Canopy strata | Species |
| --- | --- | --- |
| Dry | S1 (0 – 20 m) | *Chamaecrista scleroxylon* (Ducke) H. S. Irwin & Barneby |
|  |  | *Couratari stellata* A.C.Sm. |
|  |  | *Eschweilera coriacea* (DC.) S.A.Mori LC. |
|  |  | *Sclerolobium chrysophyllum* Poepp. & Endl. |
|  |  | *Protium apiculatum* Swart LC. |
|  |  | *Lecythis lurida* (Miers) S.A.Mori |
|  |  | *Cariocar glabrum* (Aubl.) Pers. LC. |
|  |  | *Coussarea paniculata* (Vahl) Standl. |
|  |  | *Rinorea pubiflora* (Benth.) Sprague & Sandwith |
|  |  | *Croton urucurana* Baill. |
|  |  | *Manilkara elata* (Allemão ex Miq.) Monach. |
|  |  | *Erisma uncinatum* Warm. |
|  |  | *Chimarrhis turbinata* DC. |
| Dry | S2 (20 – 40 m) | *Chamaecrista scleroxylon* (Ducke) H. S. Irwin & Barneby |
|  |  | *Manilkara elata* (Allemão ex Miq.) Monach. |
|  |  | *Lecythis lurida* (Miers) S.A.Mori |
|  |  | *Aspidosperma auriculatum* Markgr. |
|  |  | *Erisma uncinatum* Warm. |
|  |  | *Couratari stellata* A.C.Sm. |
|  |  | *Cariocar glabrum* (Aubl.) Pers. LC. |
|  |  | *Eschweilera coriacea* (DC.) S.A.Mori LC. |
|  |  | *Chimarrhis turbinata* DC. |
|  |  | *Mezilaurus itauba* (Meisn.) Taub. ex Mez. |
|  |  | *Qualea grandiflora* Mart. |
|  |  | *Hymenaea Courbaril* L. |
|  |  | *Endopleura uchi* (Huber) Cuatrec. |
|  |  | *Sclerolobium chrysophyllum* Poepp. & Endl. |
| Dry | S3 (> 40 m) | *Aspidosperma auriculatum* Markgr. |
|  |  | *Erisma uncinatum* Warm. |
|  |  | *Cariocar glabrum* (Aubl.) Pers. LC. |
|  |  | *Manilkara elata* (Allemão ex Miq.) Monach. |
|  |  | *Hymenaea Courbaril* L. |
|  |  | *Mezilaurus itauba* (Meisn.) Taub. ex Mez. |
|  |  | *Sclerolobium chrysophyllum* Poepp. & Endl. |
|  |  | *Couratari stellata* A.C.Sm. |
|  |  | *Lecythis lurida* (Miers) S.A.Mori |
| Wet | S1 (0 – 20 m) | *Coussarea paniculata* (Vahl) Standl. |
|  |  | *Protium apiculatum* Swart LC. |
|  |  | *Chamaecrista scleroxylon* (Ducke) H. S. Irwin & Barneby |
|  |  | *Lecythis lurida* (Miers) S.A.Mori |
|  |  | *Erisma uncinatum* Warm. |
|  |  | *Rinorea pubiflora* (Benth.) Sprague & Sandwith |
|  |  | *Endopleura uchi* (Huber) Cuatrec. |
|  |  | *Chimarrhis turbinata* DC. |
|  |  | *Manilkara elata* (Allemão ex Miq.) Monach. |
|  |  | *Couratari stellata* A.C.Sm. |
|  |  | *Cariocar glabrum* (Aubl.) Pers. LC. |
| Wet | S2 (20 – 40 m) | *Manilkara elata* (Allemão ex Miq.) Monach. |
|  |  | *Erisma uncinatum* Warm. |
|  |  | *Chimarrhis turbinata* DC. |
|  |  | *Chamaecrista scleroxylon* (Ducke) H. S. Irwin & Barneby |
|  |  | *Eschweilera coriacea* (DC.) S.A.Mori LC. |
|  |  | *Coussarea paniculata* (Vahl) Standl. |
|  |  | *Mezilaurus itauba* (Meisn.) Taub. ex Mez. |
|  |  | *Sclerolobium chrysophyllum* Poepp. & Endl. |
| Wet | S3 (> 40 m) | *Manilkara elata* (Allemão ex Miq.) Monach. |
|  |  | *Couratari stellata* A.C.Sm. |
|  |  | *Erisma uncinatum* Warm. |
|  |  | *Mezilaurus itauba* (Meisn.) Taub. ex Mez. |

**Table S2** Variance inflation factor (VIF) for the predictors of generalized mixed models (GMMs) developed for each quantum yield.

| Parameter | Season | Predictor | All | 0 - 20 m | 20 - 40 m | > 40 m |
| --- | --- | --- | --- | --- | --- | --- |
| ΦNPQ | Dry | PAR | 1.24 | 1.11 | 1.26 | 1.05 |
|  |  | VPD | 1.38 | 1.27 | 1.31 | 1.01 |
|  |  | Height | 1.31 | 1.19 | 1.06 | 1.06 |
|  | Wet | PAR | 1.32 | 1.08 | 1.46 | 1.24 |
|  |  | VPD | 1.38 | 1.07 | 1.45 | 1.22 |
|  |  | Height | 1.07 | 1.01 | 1.02 | 1.06 |
| ΦNO | Dry | PAR | 1.24 | 1.11 | 1.26 | 1.05 |
|  |  | VPD | 1.39 | 1.27 | 1.31 | 1.01 |
|  |  | Height | 1.32 | 1.19 | 1.06 | 1.06 |
|  | Wet | PAR | 1.32 | 1.09 | 1.47 | 1.24 |
|  |  | VPD | 1.38 | 1.07 | 1.45 | 1.22 |
|  |  | Height | 1.07 | 1.02 | 1.02 | 1.06 |
| ΦPSII | Dry | PAR | 1.24 | 1.11 | 1.26 | 1.05 |
|  |  | VPD | 1.39 | 1.28 | 1.31 | 1.01 |
|  |  | Height | 1.32 | 1.20 | 1.06 | 1.06 |
|  | Wet | PAR | 1.32 | 1.08 | 1.47 | 1.24 |
|  |  | VPD | 1.38 | 1.07 | 1.45 | 1.22 |
|  |  | Height | 1.07 | 1.01 | 1.02 | 1.07 |

**Table S3** Conditional and marginal R² values from the Generalized Mixed Models (GMMs) developed for each quantum yield across canopy strata and seasons.

| Strata | Season | Parameter | Marginal R² | Conditional R² |
| --- | --- | --- | --- | --- |
| S1 (0 – 20 m) | wet | ΦPSII | 0.82 | 0.88 |
|  |  | ΦNO | 0.57 | 0.62 |
|  |  | ΦNPQ | 0.84 | 0.88 |
| S1 (0 – 20 m) | dry | ΦPSII | 0.80 | 0.83 |
|  |  | ΦNO | 0.23 | 0.40 |
|  |  | ΦNPQ | 0.82 | 0.86 |
| S2 (20 – 40 m) | wet | ΦPSII | 0.84 | 0.88 |
|  |  | ΦNO | 0.56 | 0.65 |
|  |  | ΦNPQ | 0.80 | 0.86 |
| S2 (20 – 40 m) | dry | ΦPSII | 0.81 | 0.89 |
|  |  | ΦNO | 0.33 | 0.65 |
|  |  | ΦNPQ | 0.70 | 0.88 |
| S3 (> 40 m) | wet | ΦPSII | 0.82 | 0.90 |
|  |  | ΦNO | 0.27 | 0.64 |
|  |  | ΦNPQ | 0.71 | 0.88 |
| S3 (> 40 m) | dry | ΦPSII | 0.83 | 0.86 |
|  |  | ΦNO | 0.27 | 0.61 |
|  |  | ΦNPQ | 0.70 | 0.8 |

**Table S4** Hierarchical partitioning of explained deviance for Generalized Additive Mixed Models (GAMMs), showing the individual contributions of each predictor to the total explained deviance (Dev. explained) for each model.

| Parameter | Predictors | Dev. explained | Percentage (%) |
| --- | --- | --- | --- |
| ΦNO | PAR (by season) | 0.318 | 47.22 |
|  | VPD (by Season) | 0.160 | 23.73 |
|  | Height (by Season) | 0.049 | 7.29 |
|  | Season | 0.016 | 2.42 |
|  | Species (random effect) | 0.130 | 19.34 |
|  | Total | 0.673 | 100 |
| ΦNPQ | PAR (by season) | 0.589 | 66.67 |
|  | VPD (by Season) | 0.123 | 13.95 |
|  | Height (by Season) | 0.068 | 7.66 |
|  | Season | 0.027 | 2.96 |
|  | Species (random effect) | 0.077 | 8.76 |
|  | Total | 0.884 | 100 |
| ΦPSII | PAR (by season) | 0.677 | 74.03 |
|  | VPD (by Season) | 0.092 | 10.04 |
|  | Height (by Season) | 0.059 | 6.43 |
|  | Season | 0.013 | 1.42 |
|  | Species (random effect) | 0.074 | 8.08 |
|  | Total | 0.915 | 100 |

**Table S5** Outputs of generalized additive models (GAMs) smooth terms for the relationships between NPQt and qL (responses) with PAR and VPD (predictors) across seasons and canopy strata. The table shows the deviance explained (Dev. explained) and the effective degrees of freedom (edf) estimated for each model: edf = 1 indicates a linear relationship, and edf > 1 indicates a non-linear relationship. Significance levels are denoted as: **p < 0.001, and *p < 0.05.

| Season | Strata | Response | Predictor | Dev. explained | edf | *p*-value |
| --- | --- | --- | --- | --- | --- | --- |
| Dry | All | NPQt | PAR | 0.63 | 1.98 | < 0.001** |
|  | All | qL | PAR | 0.41 | 1.99 | < 0.001** |
|  | All | NPQt | VPD | 0.20 | 1.98 | < 0.001** |
|  | All | qL | VPD | 0.02 | 1.95 | < 0.001** |
|  | S1 | NPQt | PAR | 0.69 | 1.53 | < 0.001** |
|  | S1 | qL | PAR | 0.40 | 1.98 | < 0.001** |
|  | S1 | NPQt | VPD | 0.007 | 1.18 | 0.07 |
|  | S1 | qL | VPD | 0.06 | 1.90 | < 0.001** |
|  | S2 | NPQt | PAR | 0.63 | 1.49 | < 0.001** |
|  | S2 | qL | PAR | 0.42 | 1.99 | < 0.001** |
|  | S2 | NPQt | VPD | 0.22 | 1.94 | < 0.001** |
|  | S2 | qL | VPD | 0.02 | 1.00 | 0.17 |
|  | S3 | NPQt | PAR | 0.40 | 1.88 | < 0.001** |
|  | S3 | qL | PAR | 0.49 | 1.92 | < 0.001** |
|  | S3 | NPQt | VPD | 0.02 | 1.00 | < 0.05* |
|  | S3 | qL | VPD | 0.01 | 1.00 | < 0.05* |
| Wet | All | NPQt | PAR | 0.66 | 1.97 | < 0.001** |
|  | All | qL | PAR | 0.45 | 1.98 | < 0.001** |
|  | All | NPQt | VPD | 0.18 | 1.97 | < 0.001** |
|  | All | qL | VPD | 0.03 | 1.92 | < 0.001** |
|  | S1 | NPQt | PAR | 0.66 | 1.95 | < 0.001** |
|  | S1 | qL | PAR | 0.43 | 1.92 | < 0.001** |
|  | S1 | NPQt | VPD | 0.04 | 1.93 | < 0.001** |
|  | S1 | qL | VPD | 0.10 | 1.00 | < 0.001** |
|  | S2 | NPQt | PAR | 0.72 | 1.77 | < 0.001** |
|  | S2 | qL | PAR | 0.45 | 1.97 | < 0.001** |
|  | S2 | NPQt | VPD | 0.23 | 1.72 | < 0.001** |
|  | S2 | qL | VPD | 0.003 | 1.30 | 0.59 |
|  | S3 | NPQt | PAR | 0.56 | 1.00 | < 0.001** |
|  | S3 | qL | PAR | 0.64 | 1.97 | < 0.001** |
|  | S3 | NPQt | VPD | 0.19 | 1.62 | < 0.001** |
|  | S3 | qL | VPD | 0.11 | 1.00 | < 0.001** |

**Methods S1** Generalized additive models (GAMs) fitted to the relationships between qL and NPQt with PAR and VPD across canopy strata.

We applied a data filtering to ensure the consistency of qL and NPQt values by removing observations where qL was greater than 1, and excluding statistical outliers in NPQt using the “outliers” package in R, which eliminates values outside the interquartile range. After this filtering, the final dataset comprised 2678 measurements. GAMs with k=5 basis functions were fitted separately for qL and NPQt as response variables in each season, with smooth terms adjusted for PAR and VPD in different models, considering both the entire forest and specific canopy strata. In total, 32 models were fitted for each season. To determine the best distribution for qL and NPQt, we tested GAMs using beta regression with identity and logit links, as well as a Gaussian distribution. Model diagnostics—including QQ-plots, residual analyses, and AIC comparisons—indicated that the Gaussian model provided the best fit for both response variables. Additional validation through mean squared error (MSE) and adjusted R² comparisons confirmed the suitability of the Gaussian model for our dataset.
